# The serial mediating roles of physical activity and anger rumination in the relationship between ego depletion and cyberaggression among college students

**DOI:** 10.3389/fpsyg.2026.1922773

**Published:** 2026-10-13

**Authors:** Chao Sun, Zhonggen Yin, Caifeng Li, Yan Du, Chen Xu, Chenxi Zhang, Yijun He, Dianyuan Xue, Meng Luo

**Affiliations:** 1 School of Sports Training, Chengdu Sport University, Chengdu, Sichuan, China; 2 College of Physical Education and Health Management, Chongqing University of Education, Nanan, Chongqing, China; 3 School of Smart Business, Chongqing College of Architecture and Technology, Chongqing, China; 4 Police Physical Training Department, Sichuan Vocational College of Judicial Police, Deyang, Sichuan, China

**Keywords:** anger rumination, college students, cyberaggression, ego depletion, physical activity

## Abstract

**Objective:**

The General Aggression Model proposes that input factors influence aggressive outcomes through cognitive and emotional processing, yet most existing research has focused on pairwise relationships or parallel mediation models without testing the sequential pathways among variables. This study aimed to test a serial mediation model examining whether ego depletion is associated with cyberaggression through the sequential mediating roles of physical activity and anger rumination.

**Methods:**

A cross-sectional design was adopted. A total of 1,016 Chinese college students (aged 18–24 years) were recruited through stratified sampling. Standardized self-report scales were used to assess ego depletion, physical activity volume, anger rumination, and cyberaggression. Controlling for gender and academic grade, serial mediation pathways were evaluated using the PROCESS macro with bootstrap resampling (5,000 iterations).

**Results:**

The total effect of ego depletion on cyberaggression was significant (*b* = 0.522, *p* < 0.001), with a significant direct effect remaining after incorporating mediators (*b* = 0.240, *p* < 0.001). Mediation analysis indicated that physical activity and anger rumination partially mediated this relationship through three distinct indirect pathways: (1) physical activity alone (effect = 0.084, *p* < 0.001), (2) anger rumination alone (effect = 0.169, *p* < 0.001), and (3) the sequential pathway of physical activity followed by anger rumination (effect = 0.028, *p* < 0.001). The total indirect effect was 0.282, accounting for 54.02% of the total effect. Bootstrap 95% confidence intervals for all indirect pathways excluded zero.

**Conclusion:**

Ego depletion is both directly and indirectly associated with cyberaggression through the independent roles of physical activity and anger rumination, as well as their sequential combination. These findings highlight the value of addressing both self-regulatory depletion and cognitive-emotional processing in understanding online aggressive behaviors among university students.

## 1 Introduction

Cyberaggression among college students has become increasingly prevalent and continues to attract research attention (Souza et al., 2024; Mccomas and Gil-Rivas, 2024; Tonglin et al., 2024). The contributing factors are complex, involving the interplay of individual psychological states (Mccomas and Gil-Rivas, 2024), behavioral patterns (Johnson et al., 2022), and cognitive processes (Trifiletti et al., 2022). Ego depletion is considered an important variable associated with cyberaggression (Stucke and Baumeister, 2006). It refers to a state in which an individual's self-control resources are consumed, which is characterized by a reduced capacity for self-regulation (Baumeister et al., 1998). Physical activity, as a daily behavioral routine, is associated with individuals' emotional regulation and behavioral tendencies (Zhang et al., 2024). Anger rumination is a cognitive tendency in which individuals repeatedly dwell on angry experiences and their antecedents and consequences (Sukhodolsky et al., 2001). Previous studies have separately examined the relationships of physical activity (Yu et al., 2024), anger rumination (Alimohammadi Maki and Mansouri, 2025), and ego depletion (Stucke and Baumeister, 2006) with aggressive behavior.

The General Aggression Model provides an integrative theoretical framework for understanding the process of aggressive behavior (Kowalski et al., 2014). According to this model, aggressive behavior results from the joint influence of individual factors, situational factors, and cognitive and emotional processes. Input variables are associated with behavioral outcomes through cognitive and emotional processing pathways (Anderson and Bushman, 2002). Within this Input-Process-Output (I-P-O) framework, ego depletion is positioned as the personal/situational input factor-a state of depleted self-regulatory resources. Physical activity is conceptualized as a behavioral regulatory input that influences internal states, while anger rumination belongs to the core component of cognitive-emotional processing at the process stage. Cyberaggression represents the behavioral output. This staging suggests a theoretically specific transmission order: ego depletion → physical activity → anger rumination → cyberaggression.

Few studies have examined ego depletion, physical activity, anger rumination, and cyberaggression within a unified serial model. Most existing research has focused on testing pairwise relationships or parallel mediation effects (Sun et al., 2024; Zhao et al., 2025), with less attention paid to the possible sequential relationships among these variables. Adopting a serial mediation model allows for testing whether indirect effects are transmitted through a specific order, thereby providing a more precise account of the theoretical logical sequence among variables. Therefore, this study aims to construct a sequential mediation model to examine whether ego depletion is theoretically associated with cyberaggression through the sequential indirect pathway of physical activity and anger rumination. In addition, this study will test the separate indirect effects of physical activity and anger rumination, respectively, in order to compare the relative contributions of different pathways. Testing this sequential pathway has both theoretical and practical implications. At the theoretical level, the confirmation of a serial mediation would extend the application of the General Aggression Model to the domain of cyberaggression. At the practical level, the findings may provide theoretical reference directions for the prevention of cyberaggression among college students.

## 2 Literature review and hypothesis development

### 2.1 Ego depletion and cyberaggression

Ego depletion refers to a state in which an individual's self-control resources are temporarily consumed after engaging in self-control activities, which manifests as a reduced capacity for subsequent self-control (Baumeister et al., 1998). In the online environment, college students face various conflict situations that require self-control, all of which may consume their self-control resources. When self-control resources are at a lower level, individuals tend to demonstrate a weakened ability to inhibit impulsive behaviors (Dewall et al., 2011).

Cyberaggression refers to intentional harmful behaviors carried out by individuals through online platforms, including verbal attacks, relational aggression, and deceptive behaviors (Grigg, 2010). The anonymity, immediacy, and wide reach of the online environment may reduce individuals' perception of the consequences of their actions, thus being associated with the occurrence of aggressive behaviors (Juliati et al., 2025).

The General Aggression Model provides theoretical support for understanding the relationship between these two variables. According to this model, individual and situational factors are associated with behavioral outcomes through cognitive and emotional processing pathways (Anderson and Bushman, 2002). Ego depletion can be viewed as an individual-level input factor (Baumeister et al., 1998). When individuals are in a state of higher ego depletion, their cognitive appraisal and emotion regulation abilities may be constrained, making it more difficult for them to make rational judgments in conflict situations, which is associated with a higher likelihood of aggressive behaviors (Denson et al., 2012). Existing empirical studies support this association, finding that individuals with higher levels of ego depletion exhibit higher levels of aggressive tendencies in both laboratory tasks and online environments (Dewall et al., 2011; Osgood and Muraven, 2016). Therefore, we proposed Hypothesis H1: Ego depletion is positively associated with cyberaggression.

### 2.2 Ego depletion, physical activity, and anger rumination

Physical activity refers to regular bodily activities that individuals engage in to maintain or promote physical health (Caspersen et al., 1985). Anger rumination refers to the cognitive tendency to repeatedly think about angry experiences and their antecedents and consequences (Sukhodolsky et al., 2001). The General Aggression Model posits that input factors are associated with behavioral outcomes through cognitive and emotional processing pathways (Anderson and Bushman, 2002). Within this framework, physical activity can be viewed as a behavioral input that influences cognitive and emotional states (Ortiz-Franco et al., 2024), while anger rumination belongs to the core component of cognitive-emotional processing (Salguero et al., 2020).

Regarding the relationship between ego depletion and physical activity, existing studies have shown a negative association between the two. Maintaining regular physical activity requires sustained self-control, such as overcoming inertia and adhering to plans, all of which consume self-control resources (Hagger et al., 2010b; Su et al., 2025). When individuals are in a state of ego depletion, the available self-control resources decrease, which may make it difficult for them to maintain regular physical activity (Hagger et al., 2010a). Empirical studies have also found that individuals with lower levels of self-control resources tend to have relatively lower participation in physical activity (Boat and Cooper, 2019; Chen et al., 2024; Mavibaş et al., 2025).

Regarding the relationship between physical activity and anger rumination, existing evidence indicates a negative association between the two. Regular physical activity may reduce the time individuals spend repeatedly dwelling on angry events by providing opportunities for attention diversion (Schmitter et al., 2023). In addition, physical activity is associated with positive emotional experiences, which may reduce individuals' tendency to repeatedly think about negative events (Schmitter et al., 2023). Studies have found that individuals with higher physical activity frequency tend to have relatively lower anger rumination tendencies (Boraita et al., 2025; Mahon et al., 2025).

Furthermore, a positive association exists between ego depletion and anger rumination (Plessen et al., 2023). When individuals have insufficient self-control resources, their ability to inhibit the recurrent emergence of negative cognitions may decline, making them more susceptible to repeatedly dwelling on angry events (Jiang et al., 2025). Anger rumination, in turn, may be associated with aggressive behavior through impairing self-regulation and self-control (Alimohammadi Maki and Mansouri, 2025).

Therefore, we proposed Hypothesis H2: Physical activity mediates the relationship between ego depletion and cyberaggression.

### 2.3 Ego depletion, anger rumination, and cyberaggression

Anger rumination refers to the cognitive tendency to repeatedly think about angry experiences and their antecedents and consequences (Sukhodolsky et al., 2001). This repetitive cognitive processing may prolong individuals' negative emotional experiences (Bushman, 2002; Simons et al., 2017) and enhance their attentional bias toward provocative cues (Kaiser et al., 2018). In online interaction contexts, individuals with higher anger rumination tendencies may be more sensitive to the negative meanings in others' verbal messages (Jiang et al., 2025), which is associated with aggressive responses (García-Sancho et al., 2014; Alimohammadi Maki and Mansouri, 2025). The General Aggression Model considers cognitive and emotional processing pathways as key links between input factors and behavioral outcomes. Within this framework, anger rumination can be positioned as a typical cognitive processing process (Anderson and Bushman, 2002).

Regarding the relationship between anger rumination and aggressive behavior, existing research has provided relatively consistent evidence. Studies have shown that individuals with higher anger rumination tendencies exhibit higher levels of aggressive behavior in both laboratory tasks and self-report measures (Navas-Casado et al., 2023; Qu et al., 2024). Other studies have found that anger rumination explains individual differences in aggressive behavior, and that even after controlling for trait anger levels, anger rumination remains independently associated with aggressive behavior (Salguero et al., 2020). In terms of cyberaggression, research has indicated that anger rumination is positively associated with behaviors such as cyberbullying and online verbal aggression (Zsila et al., 2019; Camacho et al., 2021).

Regarding the relationship between ego depletion and anger rumination, this has been discussed in Section 2.2. When individuals are in a state of ego depletion, their ability to inhibit the recurrent emergence of negative cognitions may decline, making them more susceptible to repeatedly dwelling on angry events (White and Turner, 2014). Taken together, ego depletion may be associated with increased anger rumination, and increased anger rumination is in turn associated with the frequency of cyberaggression (Camacho et al., 2021). This suggests that anger rumination may play an indirect association role between ego depletion and cyberaggression. However, few empirical studies have directly tested this indirect pathway.

Based on the above theoretical reasoning and empirical findings, we proposed Hypothesis H3: Anger rumination mediates the relationship between ego depletion and cyberaggression.

### 2.4 Ego depletion, physical activity, anger rumination, and cyberaggression

The preceding sections have discussed the direct association between ego depletion and cyberaggression, as well as the separate indirect pathways through physical activity and anger rumination. However, these three variables may not function independently. The General Aggression Model posits that input factors are associated with behavioral outcomes through cognitive and emotional processing pathways (Anderson and Bushman, 2002). Within this theoretical framework, different input factors may have sequential effects rather than merely existing in parallel. This implies that the indirect association between ego depletion and cyberaggression may be realized through a sequential process involving multiple steps, in which physical activity and anger rumination assume different roles at different stages.

Specifically, ego depletion may first be associated with reduced participation in physical activity (Englert and Rummel, 2016). Physical activity is in turn negatively associated with anger rumination tendencies (Liu et al., 2023), as regular physical activity may provide individuals with opportunities to divert attention and regulate emotions (Potoczny et al., 2025). Increased anger rumination tendencies are ultimately associated with the frequency of cyberaggression (Sulistyowati et al., 2024; Ding et al., 2025). This reasoning suggests that there may be a sequential indirect pathway from ego depletion through physical activity and then through anger rumination to cyberaggression, rather than an association operating through a single mediator alone.

The theoretical basis for this sequential pathway can be found in the General Aggression Model. The model divides the process leading to aggressive behavior into multiple stages, emphasizing that variables at different stages have progressive relationships. Ego depletion belongs to the input-stage individual factor, physical activity can be viewed as a behavioral input that influences internal states, anger rumination belongs to the cognitive-emotional process at the processing stage, and cyberaggression is the outcome variable at the output stage. This staging suggests that there may be a theoretically specific transmission order among these variables.

Existing research provides indirect evidence for this sequential pathway. Studies have found that ego depletion is negatively correlated with physical activity participation, other studies have indicated that physical activity is negatively correlated with anger rumination, and still others have shown that anger rumination is positively correlated with cyberaggression. Although these findings are independent, they collectively suggest a possible sequential pathway. However, no studies have yet included these four variables in the same serial model for examination.

Therefore, we proposed Hypothesis H4: Physical activity and anger rumination play a serial mediating role in the relationship between ego depletion and cyberaggression (ego depletion → physical activity → anger rumination → cyberaggression) (Figure 1). It should be noted that the directional arrows in this hypothesis represent the theoretical ordering derived from the General Aggression Model's stage-based framework, not proven causal sequences. Given the cross-sectional design of this study, these arrows should be interpreted as statistical pathways rather than causal mechanisms. Causal inferences would require further examination through longitudinal or experimental designs.

**Figure 1:**
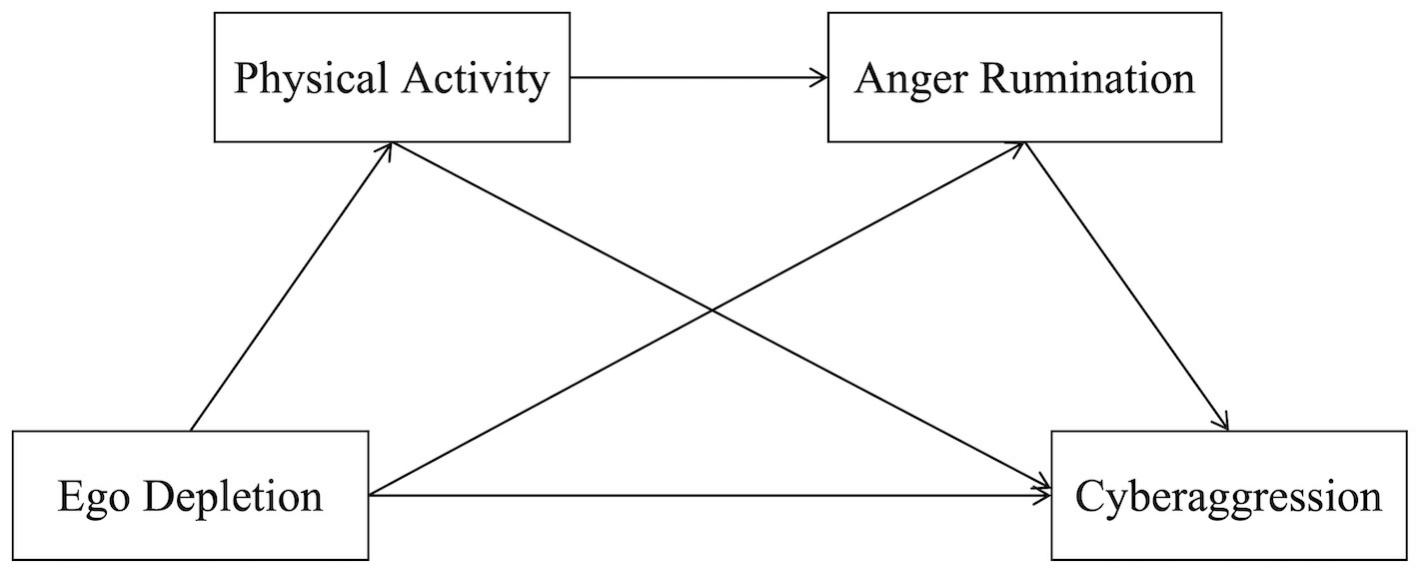
Conceptual model of the serial mediation association.

## 3 Methods

### 3.1 Procedures and participants

#### 3.1.1 Participants and procedure

A cross-sectional design was adopted. From December 5 to December 31, 2025, a stratified sampling method was used to recruit 1,100 college students from 11 provinces across five regions of China (eastern, central, western, southern, and northern). Physical education teachers distributed the questionnaires. Before distribution, the research team provided special training to these teachers on key aspects of the questionnaire design, including voluntary participation procedures, withdrawal mechanisms, and confidentiality requirements.

Afterwards, the teachers administered the questionnaires to the students. Participants completed the questionnaires under the teachers' supervision. During the process, the teachers explained the voluntary nature of participation, the meaning of the questions, and the right to withdraw. After data collection, 84 invalid responses were excluded based on pre-established criteria: (1) completion time that was too short or too long; and (2) inattentive response patterns (e.g., repetitive or contradictory answers).

The final sample consisted of 1,016 participants, yielding an effective response rate of 84.67%. Participants' ages ranged from 18 to 24 years (M = 19.5, SD = 1.355). There were 564 males (55.51%) and 452 females (44.49%). The grade distribution was as follows: 376 freshmen (37.01%), 329 sophomores (32.38%), 206 juniors (20.28%), and 105 seniors (10.33%). In terms of household registration, 487 participants (47.93%) were from urban areas and 529 (52.07%) from rural areas. Exclusion criteria included: (1) severe physical illness; (2) history of mental disorder; and (3) receipt of psychological treatment within the past 3 months.

Data were collected via an online platform (https://www.wenjuan.com/s/UZBZJvYWmq/#) using an electronic questionnaire consisting of five parts: demographic information (age, grade, gender, household registration type, etc.), the Ego Depletion Scale, the Physical Activity Scale, the Anger Rumination Scale, and the Cyberaggression Scale. All participants provided electronic informed consent before data collection. After data collection, all information was anonymized to ensure confidentiality. The anonymized data set will be stored according to institutional ethical guidelines and data management policies, and will be available for research purposes for at least 5 years after publication. Qualified researchers may apply for access to the data, but must sign a formal data-sharing agreement to ensure participant privacy and compliance with the original informed consent terms.

### 3.2 Ethics approval and consent to participate

This study strictly followed the ethical principles of the Declaration of Helsinki. The research protocol was approved by the Ethics Committee of Chongqing University of education (approval number: CQUE-LL20251205021). All participants provided electronic informed consent via the online platform before formal participation. The informed consent form detailed the study purpose, voluntary participation, confidentiality agreements, and the right to withdraw at any time without consequence. To protect participant privacy, all data were anonymized during collection and analysis, and no personally identifiable information was retained in the final data set.

### 3.3 Measures

#### 3.3.1 Ego depletion scale

Ego depletion was measured using the Ego Depletion Scale developed by Lin (Lin and Johnson, 2015). This unidimensional scale consists of five items (e.g., “It would take a lot of effort for me to concentrate on something right now”). Items are rated on a 7-point Likert scale (1 = strongly disagree, 7 = strongly agree). Higher scores indicate greater ego depletion. In this study, Cronbach's alpha was 0.916. The confirmatory factor analysis showed: χ^2^/df = 2.116, CFI = 0.996, GFI = 0.996, RFI = 0.986, RMSEA = 0.033.

#### 3.3.2 Physical activity scale

Physical activity was assessed using the self-report Physical Activity Rating Scale (PAR-3) revised by Deqing (1994), which measures participants' self-reported physical activity over the past month. The scale includes three factors: exercise intensity, exercise duration, and exercise frequency. It uses a 5-point scoring method. The total physical activity score was calculated using the formula: activity volume = intensity × (duration−1) × frequency. Higher scores indicate larger amounts of physical activity per session. In this study, Cronbach's alpha was 0.842.

#### 3.3.3 Anger rumination scale

Anger rumination was measured using the Anger Rumination Scale developed by Sukhodolsky et al. (2001). This scale includes 19 items across four dimensions: angry afterthoughts (6 items, e.g., “I keep thinking about an angry experience”), revenge fantasies (4 items, e.g., “After a conflict, I have revenge fantasies for a long time”), angry memories (5 items, e.g., “I think about events that made me angry for a long time”), and understanding causes (4 items, e.g., “I analyze the events that make me angry”). Items are rated on a 4-point scale (1 = never, 4 = always). Higher scores indicate greater agreement with the item descriptions. In this study, Cronbach's alpha was 0.962. The confirmatory factor analysis showed: χ^2^/df = 1.765, CFI = 0.978, GFI = 0.974, RFI = 0.943, RMSEA = 0.027.

#### 3.3.4 Cyberaggression scale

Cyberaggression was measured using the Cyberaggression Scale developed by Little et al. (2003). This scale consists of two subscales with a total of four dimensions and 31 items. The instrumental aggression subscale includes two dimensions with 15 items, and the reactive aggression subscale includes two dimensions with 16 items. Each item is rated on a 4-point scale (1 = never, 4 = always). Higher scores indicate that the item descriptions are more consistent with the participants' own behavior. In this study, Cronbach's alpha was 0.980. The confirmatory factor analysis showed: χ^2^/df = 1.448, CFI = 0.987, GFI = 0.979, RFI = 0.925, RMSEA = 0.022.

### 3.4 Statistical analysis methods

Data analysis proceeded in three steps.

First, common method bias was assessed using Harman's single-factor test.

Second, descriptive statistics and correlational analyses were performed. For all variables (ego depletion, physical activity, anger rumination, and cyberaggression), means, standard deviations, and Pearson correlation coefficients were calculated.

Third, a serial mediation analysis was conducted using the PROCESS macro (version 4.0) for SPSS 25.0. A sequential mediation model was constructed to examine the pathways from ego depletion to cyberaggression through physical activity and anger rumination. Gender and grade were included as covariates. The bootstrap resampling method (5,000 iterations) with 95% confidence intervals was used to test the indirect effects. The PROCESS macro (Model 6) is specifically designed for testing serial mediation models with two mediators in sequence, providing bootstrap confidence intervals for indirect effects while controlling for covariates.

## 4 Results

### 4.1 Common method bias test

Since all data were collected through self-report questionnaires, Harman's single-factor test was conducted to assess potential common method bias. An exploratory factor analysis was performed on all items from the Ego Depletion Scale, Physical Activity Scale, Anger Rumination Scale, and Cyberaggression Scale. Principal component analysis was used to extract common factors, and the first unrotated factor was examined (Podsakoff et al., 2003). The results showed that the first factor accounted for 37.966% of the variance, which is below the critical threshold of 40%, indicating that common method bias was not a significant concern in this study. However, it should be noted that Harman's single-factor test is widely considered a relatively lenient method for detecting common method bias. While this test provided preliminary evidence that common method bias is unlikely to be a major threat to the validity of our findings, future research should employ more rigorous approaches such as marker variable techniques, multi-source data collection (e.g., peer reports or behavioral logs), or longitudinal designs to more robustly control for common method variance.

### 4.2 Descriptive statistics and correlations

Descriptive statistics and Pearson correlation analyses were conducted for the four variables (ego depletion, physical activity, anger rumination, and cyberaggression), as shown in Table 1, All variables demonstrated significant intercorrelations.

**Table 1:** Descriptive statistics and correlation analysis of variables.

Variable	Mean	Standard deviation	Ego depletion	Physical activity	Anger rumination	Cyber aggression
Ego depletion	20.01	5.300	1			
Physical activity	19.42	16.653	−0.184^**^	1		
Anger rumination	42.67	9.637	0.271^**^	−0.252^**^	1	
Cyber aggression	51.10	14.887	0.186^**^	−0.244^**^	0.323^**^	1

^**^ *p* < 0.01.

Ego depletion was positively correlated with cyberaggression (*r* = 0.186, *p* < 0.01), negatively correlated with physical activity (*r* = −0.184, *p* < 0.01), and positively correlated with anger rumination (*r* = 0.271, *p* < 0.01). Physical activity was negatively correlated with anger rumination (*r* = −0.252, *p* < 0.01) and with cyberaggression (*r* = −0.244, *p* < 0.01). Anger rumination was positively correlated with cyberaggression (*r* = 0.323, *p* < 0.01).

### 4.3 Mediating association analysis

To examine the statistical associations of ego depletion, physical activity, and anger rumination on cyberaggression, hierarchical regression analyses were conducted. Ego depletion, physical activity, and anger rumination were entered as independent variables, with cyberaggression as the dependent variable (see Table 2). The results showed that ego depletion was positively associated with cyberaggression (β = 0.186, *t* = 6.022, *p* < 0.001); physical activity was negatively associated with cyberaggression (β = −0.244, *t* = −8.002, *p* < 0.001); and anger rumination was positively associated with cyberaggression (β = 0.323, *t* = 10.873, *p* < 0.001).

**Table 2:** Regression analysis of ego depletion, physical activity, anger rumination on cyberaggression.

Predictor	Cyber aggression			
	β	***T***	***F***	***R**^2^* **adj**
Ego depletion	0.186	6.022^***^	36.268	0.034
Physical activity	−0.244	−8.002^***^	64.034	0.058
Anger rumination	0.323	10.873^***^	118.226	0.104

*R*^2^ adj is the adjusted *R*-squared value. ^***^p < 0.001.

A serial mediation model was tested using the PROCESS macro (version 4.0) for SPSS 25.0. With ego depletion (X) as the independent variable, cyberaggression (Y) as the dependent variable, and physical activity (M1) and anger rumination (M2) as mediators, we examined the serial mediating effects of physical activity and anger rumination on the relationship between ego depletion and cyberaggression, after controlling for demographic variables (gender and grade). Model 6 of the PROCESS macro was used for hypothesis testing, and the bootstrap method with 5,000 resamples and 95% confidence intervals (CIs) was applied to test the serial mediation effects.

As shown in Table 3, the total effect of ego depletion on cyberaggression was significant (effect = 0.522, 95% CI [0.352, 0.692]). The bootstrap analysis further indicated that the total indirect effect was significant (effect = 0.282, 95% CI [0.211, 0.358]), and the direct effect remained significant after controlling for the mediators (effect = 0.240, 95% CI [0.072, 0.409]), accounting for 54.023% and 45.977% of the total effect, respectively. This suggests that physical activity and anger rumination serve as partial mediators in this relationship.

**Table 3:** Mediation effect sizes and confidence intervals.

Association pathway	Association estimate	BOOT SE	BOOT LLCI	BOOT ULCI	Relative mediation association
Total association	0.522^***^	0.087	0.352	0.692	100%
Direct association	0.240^***^	0.086	0.072	0.409	45.977%
Total indirect association	0.282^***^	0.037	0.211	0.358	54.023%
Indirect association 1 (Physical activity)	0.084^***^	0.022	0.046	0.131	16.092%
Indirect association 2 (Anger rumination)	0.169^***^	0.029	0.115	0.229	32.375%
Indirect association 3 (Physical activity → Anger rumination)	0.028^***^	0.007	0.016	0.044	5.364%

Indirect association 1: Ego Depletion → Physical Activity → Cyberaggression;

Indirect association 2: Ego Depletion → Anger Rumination → Cyberaggression;

Indirect association 3: Ego Depletion → Physical Activity → Anger Rumination → Cyberaggression.

^***^p < 0.001, All coefficients reported in this table are unstandardized coefficients (b). Standardized coefficients (β) for individual paths are presented in Figure 2. Percentages are based on cross-sectional data and serve only as descriptive association size comparisons; they do not imply causal decomposition.

Specifically, the total indirect effect consisted of three pathways: Pathway 1 (ego depletion → physical activity → cyberaggression), with an effect of 0.084 (16.092%, 95% CI [0.046, 0.131]); Pathway 2 (ego depletion → anger rumination → cyberaggression), with an effect of 0.169 (32.375%, 95% CI [0.115, 0.229]); and Pathway 3 (ego depletion → physical activity → anger rumination → cyberaggression), with an effect of 0.028 (5.364%, 95% CI [0.016, 0.044]). The bootstrap 95% confidence intervals for all three indirect pathways did not contain zero, indicating that all were statistically significant indirect pathways. Among them, Pathway 2 (anger rumination alone) had the largest effect size, while the serial mediation pathway (Pathway 3) had a smaller but still significant effect, as confirmed by its bootstrap 95% confidence interval [0.016, 0.044] excluding zero.

These findings suggest that ego depletion is not only directly associated with cyberaggression, but also indirectly associated through the negative statistical association with physical activity, the positive statistical association with anger rumination, and the combined pathway involving the negative association between physical activity and anger rumination. These statistical relationships provide empirical support for the theoretical pathways among the variables, and Hypothesis 4 is supported. However, causal inferences would require further examination through longitudinal or experimental studies. Path diagrams illustrating these associations are presented in Figure 2.

**Figure 2:**
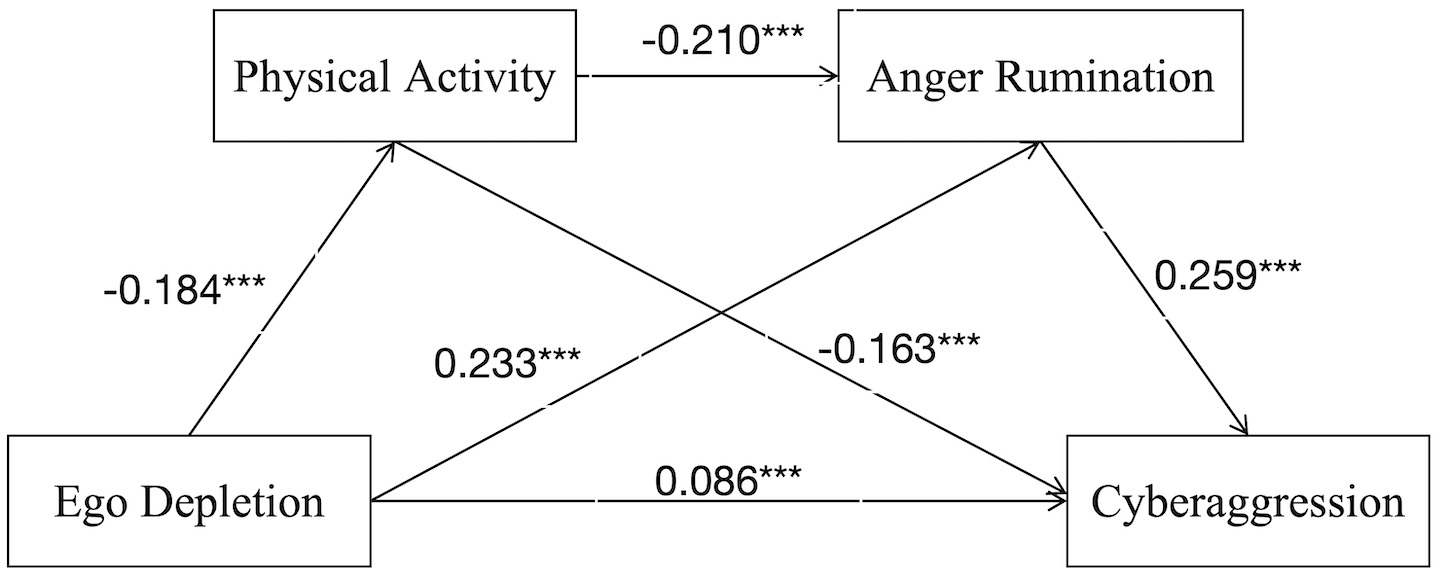
Model of serial mediating roles of physical activity and anger rumination between ego depletion and cyberaggression. Path coefficients shown are standardized (β). ***p < 0.001. Covariates: gender and grade.

## 5 Discussion

This study aimed to explore the association mechanism between ego depletion and cyberaggression among college students, with a particular focus on the potential sequential indirect pathway through physical activity and anger rumination. The findings supported the hypothesized serial mediation model, in which ego depletion is indirectly associated with cyberaggression through the sequential association of physical activity and anger rumination. The following sections discuss the main findings, theoretical contributions, practical implications, and research limitations.

### 5.1 Main findings

This study found a significant positive statistical association between ego depletion and cyberaggression among college students, consistent with previous research. Prior laboratory-based studies have shown that individuals with higher levels of ego depletion exhibit higher levels of aggressive tendencies in subsequent tasks (Dewall et al., 2007). In the domain of online behavior, studies have also indicated that ego depletion is positively associated with online verbal aggression and cyberbullying (Zhao et al., 2025). The General Aggression Model provides a theoretical explanation for this association. According to this model, individual-level input factors influence cognitive and emotional processing. Ego depletion reflects a temporary decline in self-regulatory ability, which may limit individuals' capacity for rational appraisal in online conflict situations, which is associated with aggressive responses (Zhao et al., 2025). The present study confirmed this association in a college student sample, further supporting the explanatory relevance of ego depletion as a factor associated with cyberaggression.

Second, this study demonstrated that physical activity serves as a separate indirect pathway between ego depletion and cyberaggression. Specifically, ego depletion was negatively associated with physical activity participation, and physical activity participation was negatively associated with cyberaggression, and this indirect effect was statistically significant. Previous studies have separately reported the negative association between ego depletion and physical activity (Dorris et al., 2012; Xiang et al., 2017; Finne et al., 2019), and between physical activity and aggressive behavior (Xu et al., 2025), but few have incorporated all three variables into a single mediation model. The present study integrated these two independent lines of research into a coherent pathway, suggesting that physical activity may be one explanatory pathway in the association between ego depletion and cyberaggression. From the perspective of the General Aggression Model, physical activity can be viewed as a behavioral input that influences individuals' internal states (Qin et al., 2024), and its absence may leave individuals without effective emotional regulation channels when faced with negative stimuli (Liu et al., 2022; Wang et al., 2024).

Third, this study found that anger rumination also served as a significant separate indirect pathway between ego depletion and cyberaggression. Among the three indirect pathways, anger rumination alone had the largest effect size, accounting for 32.375% of the total indirect effect. This suggests that anger rumination may be a relatively strong explanatory factor in the association between ego depletion and cyberaggression. Previous research has indicated a stable and relatively strong association between anger rumination and aggressive behavior (Nasiri and Noury, 2021; Alimohammadi Maki and Mansouri, 2025), which persists even after controlling for trait anger (Anestis et al., 2009). The present study further found that ego depletion is associated with increased anger rumination tendencies, consistent with the theoretical expectation that individuals with insufficient self-control resources have greater difficulty inhibiting the recurrence of negative cognitions (Hagger et al., 2010a). Within the General Aggression Model framework, anger rumination belongs to the core component of cognitive processing (Jiang et al., 2025), and its bridging role between input factors and behavioral outcomes was clearly demonstrated in this study. To further understand this pattern, we considered why anger rumination accounts for a larger share of mediation than physical activity. Anger rumination operates as a more proximal cognitive process, directly generating the hostile automatic thoughts, revenge fantasies, and attentional biases that immediately precede aggressive behavior (Bushman, 2002; García-Sancho et al., 2014). In contrast, physical activity functions as a more distal behavioral buffer-it influences emotional and cognitive states indirectly through attention diversion, physiological changes, and positive affect generation (Schmitter et al., 2023)-but does not directly produce or inhibit aggressive impulses at the moment of provocation. This conceptualization aligns with the GAM's framework, in which cognitive-emotional processing (anger rumination) is positioned closer to behavioral output than behavioral inputs (physical activity) in the theoretical sequence.

The central finding of this study is the sequential indirect association through physical activity and anger rumination in the relationship between ego depletion and cyberaggression. Although the effect size of this serial pathway (5.364% of the total effect) was smaller than the two separate mediation pathways, its bootstrap confidence interval did not contain zero, indicating that this sequential indirect effect was statistically significant. This finding provides empirical support for the General Aggression Model's theoretical expectation of progressive relationships among variables at different stages, consistent with previous research. Ego depletion was first associated with reduced physical activity (Dorris et al., 2012), reduced physical activity was then associated with elevated anger rumination (Liu et al., 2023; Zhu B. et al., 2024), and elevated anger rumination was ultimately associated with higher levels of cyberaggression (García-Sancho et al., 2016; Camacho et al., 2021; Zhu W. et al., 2024). This suggests that physical activity and anger rumination are not fully parallel mediators; there may be a sequential relationship between them. Previous research has often treated physical activity and anger rumination as independent influencing factors (Liu et al., 2023; Guo et al., 2025); the present study, through a serial model, revealed the logical dependency between them, enriching the existing understanding of how these four variables interact. The modest magnitude of the serial pathway (5.36%) is expected in multi-stage mediation models, where the compound indirect effect is the product of the three coefficients forming the sequential chain, naturally yielding a smaller value than single-mediator paths. This reflects the statistical nature of sequential mediation rather than theoretical weakness. More importantly, the bootstrap confidence interval [0.016, 0.044] excluded zero, confirming its statistical reliability. The theoretical importance of the serial pathway lies in revealing the sequential logic, ego depletion → reduced physical activity → elevated anger rumination → increased cyberaggression, which supports the GAM's stage-based framework.

### 5.2 Theoretical contributions and implications

First, this study incorporated ego depletion, physical activity, anger rumination, and cyberaggression into a unified serial model, extending the analytical scope of existing literature. Previous research has largely focused on pairwise relationships or parallel mediation effects (Nasiri and Noury, 2021; Liu et al., 2023), with less attention given to placing these variables within a sequential theoretical framework. By testing three indirect pathways simultaneously in the same sample, this study found that anger rumination alone had the largest effect size, while the serial pathway, although smaller, remained statistically significant. This suggests that parallel mediation models may not fully capture the complex relationships among variables, and incorporating variables into models according to their theoretical order may facilitate a more comprehensive understanding of association mechanisms.

Second, this study extended the application of the General Aggression Model to the domain of cyberaggression research. Although the model has proposed a theoretical framework in which input factors are associated with behavioral outcomes through cognitive and emotional processing pathways, most existing empirical studies have applied it to traditional aggression or violent media effects (Kersten and Greitemeyer, 2024; Allen et al., 2018). This study positioned ego depletion as an individual-level input factor, physical activity as a behavioral input influencing internal states, and anger rumination as a core component of cognitive processing. This positioning aligns with the General Aggression Model's stage-based framework and provides empirical support for the model's applicability to cyberaggression.

Third, this study revealed a unique role of physical activity in ego depletion research. Previous studies have often treated physical activity either as a direct protective factor against aggression or as an independent health behavior variable (Park et al., 2021; Andrade et al., 2025). The serial mediation results showed that physical activity not only has a direct negative association with cyberaggression, but its negative association with anger rumination is also an important link in explaining the association between ego depletion and cyberaggression. This finding suggests that physical activity may be associated with aggression through its influence on cognitive and emotional processing, rather than solely through improved physical health, offering a new theoretical perspective for understanding the relationship between physical activity and aggressive behavior.

Fourth, this study further confirmed the central role of anger rumination in cognitive processing pathways. Among the three indirect pathways, anger rumination alone had the largest effect size and played the final role in the serial pathway. Even when ego depletion and physical activity were included in the same model, the association between anger rumination and cyberaggression remained statistically strong, indicating that anger rumination plays a key transmission role in the sequential association between ego depletion and cyberaggression.

### 5.3 Practical implications and applications

The findings of this study provide several reference directions for the prevention of cyberaggression among college students.

First, the negative statistical association between physical activity and cyberaggression, along with physical activity serving as an indirect pathway between ego depletion and cyberaggression, suggests that attending to college students' physical activity levels may be a worthwhile reference point. Universities could facilitate student participation in physical activity through course offerings, access to sports facilities, and support for sports clubs and activities.

Second, the relatively stable positive association between anger rumination and cyberaggression, along with its largest effect size among the indirect pathways, suggests that anger rumination may be a cognitive factor relatively strongly associated with cyberaggression. Mental health education programs could incorporate content on emotion regulation strategies to help students recognize and adjust cognitive patterns characterized by repeated dwelling on negative events.

Third, the serial mediation pathway suggests that physical activity and anger rumination may play different stage-based roles in the sequential association between ego depletion and cyberaggression. This implies that focusing on a single factor may not fully capture the association process; simultaneously attending to both behavioral patterns and cognitive tendencies may be more appropriate.

It should be emphasized that the above reference directions are based on statistical associations from a cross-sectional study. This study cannot answer questions of causality, and future research may further examine these possibilities through longitudinal or experimental designs. In practical applications, appropriate focal points should be selected according to specific circumstances, and the influence of individual difference factors should be fully considered.

### 5.4 Limitations and future directions

Several limitations of this study should be considered when interpreting the results.

First, this study employed a cross-sectional design, with all variables measured at a single time point. Therefore, the statistical associations among variables cannot speak to temporal order or causality. Future research could adopt longitudinal designs to measure these variables at multiple time points and examine dynamic relationships over time.

Second, all data in this study were collected through self-report from college students, which may be subject to social desirability bias, recall bias, and individual differences in interpretation. Although this study conducted Harman's single-factor test as a preliminary assessment of common method bias, this test has relatively lenient criteria, and more rigorous statistical controls were not implemented. Future research could consider incorporating multi-source data, such as peer reports, behavioral logs, or wearable devices, along with more stringent procedures for controlling common method bias.

Third, the sample was limited to Chinese college students aged 18 to 24 years. This restricts the generalizability of the findings to other age groups or cultural contexts. Future research could test the present model in more diverse populations.

Fourth, this study examined only two mediating variables—physical activity and anger rumination. Other potential mediating variables, such as social support or coping styles, were not included. Future research could extend the model to include additional relevant variables or examine moderating effects.

Fifth, physical activity was assessed using a brief scale that did not distinguish between different types of physical activities. Team sports and individual sports may differ in their effects on social interaction and emotional regulation. Future research could employ more refined measurement tools to differentiate various types of physical activity.

Sixth, the PAR-3 assesses self-reported physical activity volume and intensity through subjective ratings rather than objective tracking (e.g., accelerometry). This measurement approach may be subject to recall bias and does not capture objective physiological exertion levels. Future research could employ objective physical activity monitoring to complement self-report measures.

Seventh, the cross-sectional nature of this study prevents testing the temporal ordering implied by the General Aggression Model's stage-based framework. Although our serial model is theoretically grounded, alternative orderings of the variables cannot be ruled out with the current design.

Despite these limitations, this study revealed a pattern of statistical associations among the variables within a cross-sectional framework, providing a theoretical reference for future research.

## 6 Conclusion

Based on the General Aggression Model, this study evaluated the serial mediating mechanisms of physical activity and anger rumination in the relationship between ego depletion and cyberaggression among university students. Ego depletion demonstrated a significant direct positive association with cyberaggression, as well as indirect associations operating through three distinct pathways: independent mediation through physical activity, independent mediation through anger rumination, and sequential mediation through physical activity followed by anger rumination.

These results demonstrate that physical activity and anger rumination do not operate solely as parallel mechanisms; rather, lower physical activity engagement secondary to ego depletion can exacerbate anger rumination, which in turn relates to heightened cyberaggression. Theoretically, these findings extend the GAM to digital contexts by identifying specific behavioral-cognitive chains. Practically, interventions aimed at curbing campus cyberaggression should combine strategies that preserve self-regulatory energy, encourage regular physical activity, and mitigate cognitive anger rumination.

## Data Availability

The original contributions presented in the study are included in the article/Supplementary material, further inquiries can be directed to the corresponding authors.
